# Changes in Hepatic Gene Expression upon Oral Administration of Taurine-Conjugated Ursodeoxycholic Acid in ob/ob Mice

**DOI:** 10.1371/journal.pone.0013858

**Published:** 2010-11-05

**Authors:** Jae-Seong Yang, Jin Taek Kim, Jouhyun Jeon, Ho Sun Park, Gyeong Hoon Kang, Kyong Soo Park, Hong Kyu Lee, Sanguk Kim, Young Min Cho

**Affiliations:** 1 School of Interdisciplinary Bioscience and Bioengineering, Pohang University of Science and Technology, Pohang, South Korea; 2 Department of Internal Medicine, Seoul National University College of Medicine, Seoul, South Korea; 3 Department of Life Science, Pohang University of Science and Technology, Pohang, South Korea; 4 Department of Pathology, Seoul National University College of Medicine, Seoul, South Korea; Institute of Preventive Medicine, Denmark

## Abstract

Nonalcoholic fatty liver disease (NAFLD) is highly prevalent and associated with considerable morbidities. Unfortunately, there is no currently available drug established to treat NAFLD. It was recently reported that intraperitoneal administration of taurine-conjugated ursodeoxycholic acid (TUDCA) improved hepatic steatosis in ob/ob mice. We hereby examined the effect of oral TUDCA treatment on hepatic steatosis and associated changes in hepatic gene expression in ob/ob mice. We administered TUDCA to ob/ob mice at a dose of 500 mg/kg twice a day by gastric gavage for 3 weeks. Body weight, glucose homeostasis, endoplasmic reticulum (ER) stress, and hepatic gene expression were examined in comparison with control ob/ob mice and normal littermate C57BL/6J mice. Compared to the control ob/ob mice, TUDCA treated ob/ob mice revealed markedly reduced liver fat stained by oil red O (44.2±5.8% vs. 21.1±10.4%, *P*<0.05), whereas there was no difference in body weight, oral glucose tolerance, insulin sensitivity, and ER stress. Microarray analysis of hepatic gene expression demonstrated that oral TUDCA treatment mainly decreased the expression of genes involved in *de novo* lipogenesis among the components of lipid homeostasis. At pathway levels, oral TUDCA altered the genes regulating amino acid, carbohydrate, and drug metabolism in addition to lipid metabolism. In summary, oral TUDCA treatment decreased hepatic steatosis in ob/ob mice by cooperative regulation of multiple metabolic pathways, particularly by reducing the expression of genes known to regulate *de novo* lipogenesis.

## Introduction

Nonalcoholic fatty liver disease (NAFLD) is the most common cause of chronic liver disease [1], [2], [3] and its prevalence ranges from 10–30% of the general population in the United States [1], [3], [4], [5]. NAFLD includes a spectrum of liver diseases from simple hepatic steatosis to nonalcoholic steatohepatitis (NASH) [1], [3], where the latter is known to increase the risk of liver cirrhosis and hepatocellular carcinoma [6]. Insulin resistance and metabolic syndrome are commonly associated with NAFLD and their presence is a predictable factor of progressive liver dysfunction, which may lead to hepatic failure [7]. The pathophysiology of NAFLD is complex involving dietary factors, physical inactivity, obesity, and genetic components [1], [2], [3]. Although weight reduction by lifestyle modification (i.e., caloric restriction and increased physical activity) remains the most effective and desirable treatment of NAFLD [1], [8], [9], long-term adherence to a new lifestyle is the mainstay for success [8], which is practically very difficult to achieve.

Several agents are known to improve NAFLD histologically or biochemically in animal models and humans [1], [2], [10], [11], [12], [13]. Among them, ursodeoxycholic acid (UDCA), an endogenous bile acid, improves liver function in patients with a wide range of chronic liver diseases [14], [15], [16]. Furthermore, UDCA was demonstrated to decrease liver enzyme levels and the degree of steatosis in an open label pilot study [17]. However, in a randomized placebo-controlled trial conducted in NASH patients, UDCA revealed only comparable effects to the placebo in terms of serum liver enzyme levels, hepatic steatosis, necroinflammation, and fibrosis [12]. Taurine-conjugated UDCA (TUDCA) is more hydrophilic and has a more obvious cytoprotective effect against hepatocellular injury than UDCA [18], [19], [20]. It was reported that intraperitoneally injected TUDCA improved hepatic steatosis in ob/ob mice, which was associated with improvement of endoplasmic reticulum (ER) stress in the liver [21]. In a very recent study conducted in obese human subjects focused on tissue insulin sensitivity [22], oral TUDCA treatment did not alter intrahepatic triglyceride content. However, the baseline intrahepatic triglyceride content of the subjects in TUDCA treatment group was only modestly increased (8.2%). Therefore, it remains inconclusive whether oral administration of TUDCA reveals similar effects to parenteral administration in terms of improving hepatic steatosis. Since orally administrated TUDCA is absorbed via active transport in the terminal ileum and undergoes a significant hepatic first pass effect and enterohepatic circulation [23], [24], the working mechanism of orally administrated TUDCA may be different from that of intraperitoneally injected TUDCA [21].

We hereby investigated the effect of oral TUDCA treatment on hepatic steatosis and gene expression in ob/ob mice. To figure out the mechanism of action of TUDCA on hepatic steatosis, we systematically analyzed the microarray data. First, we verified the relevance of differentially expressed genes (DEGs) based on the preexisting literature. Second, we analyzed the expression of the genes regulating each component of lipid homeostasis (i.e., *de novo* lipogenesis, uptake, oxidation, and export). Third, we conducted gene enrichment analysis using Gene Ontology (GO) to identify the significantly altered functional groups of DEGs. Lastly, we adopted pathway analysis to elucidate the collective behavior of DEGs, which provides complementary information to conventional single gene-based analysis [25].

## Materials and Methods

### Animal experiments

The study protocol was approved by the Institutional Animal Care and Use Committee of Seoul National University Hospital. Six-week-old male ob/ob mice (C57BL/6J-ob/ob) and their control littermates (C57BL/6J) were purchased from Shizuoka Laboratory Center (Shizuoka, Japan). The ob/ob mice were given TUDCA (Tokyo Tanabe, Tokyo, Japan) at a dose of 500 mg/kg twice a day (8:00 AM and 8:00 PM) by gastric gavage for 3 weeks (OB-TUDCA group, n = 6). Given that the oral bioavailability of TUDCA is approximately 65% [23], we doubled the dose of TUDCA that used in intraperitoneal injection [21]. We administered the same volume of tap water to control ob/ob mice (OB-control group, n = 6) and normal control C57BL/6J mice (N-control group, n = 6) twice a day by gastric gavage for 3 weeks. Mice were fed a normal chow diet (Purina LabDiet, St Louis, MO, USA) *ad libitum*. Body weight and fed-state plasma glucose levels were monitored during the study period. The serum levels of aspartate aminotransferase (AST) and alanine aminotransferase (ALT) were enzymatically measured using an autoanalyzer (ADVIA 1650, Siemens Medical Solutions Diagnostics, Tarrytown, NY, USA).

At week 3, intraperitoneal glucose tolerance test (IPGTT) and insulin tolerance test (ITT) were performed according to standard methods. Briefly, for IPGTT, the mice were fasted overnight for 12 hours and glucose (0.5 g/kg) was intraperitoneally administered at 9 AM. For ITT, the mice were fasted for 6 hours from 8 AM, when drug or vehicle was administered by gavage, and then insulin (2 IU/kg) was intraperitoneally injected. Blood glucose levels were measured using a hand-held glucometer (One Touch Ultra, LifeScan, Milpitas, CA, USA).

### Histomorphometric analysis and markers of oxidative stress and ER stress

The mice were killed after an 8 hour fasting. The livers were immediately retrieved and fixed in 10% neutral buffered formalin for 24 hours or kept freshly frozen. The formalin fixed liver tissues were embedded in paraffin. We performed hematoxylin and eosin (H&E) staining on the paraffin embedded liver sections (4 µm thickness) and oil red O staining on fresh frozen liver sections (10 µm thickness). Oil red O stained liver sections were examined under a light microscope (Olympus BX 50, Tokyo, Japan) at 100× magnification, and the images were analyzed by Image J software (National Institutes of Health, Bethesda, MD, USA). The hepatic lipid content is presented as the percentage of oil red O stained area per total liver parenchymal area in a given section. Markers for ER stress were analyzed by Western blotting and reverse transcription PCR (detailed methods are shown in Text S1). To assess oxidative stress in the liver, we stained 4-hydroxy-2-nonenal (4-HNE), a marker of lipid peroxidation, with a mouse anti-4-HNE antibody (Abcam Inc., Cambridge, MA), and measured the brown color intensity of 20 different regions with Image J.

### Statistics

All data except the microarray results are presented as the mean ± S.E.M. Using a commercial software (Prism 5.0, GraphPad, San Diego, CA, USA), we performed t-test, Kruskal-Wallis test, and Dunn's multiple comparison test, where appropriate. A *P*<0.05 was considered to be statistically significant.

### Microarray

Isolation of total RNA from the liver tissue was performed using the RNeasy Micro Kit (Qiagen, Valencia, CA, USA) according to the manufacturer's instructions. Probes for the GeneChip® Mouse Gene 1.0 ST Array (Affymetrix, Santa Clara, CA, USA) were prepared and hybridized using the GeneChip Whole Transcript Sense Target Labeling Assay (Affymetrix) and scanned according to the manufacturer's instructions (Scanner 3000 7G, Affymetrix). Methods for DEG selection and GO enrichment analysis are shown in Text S1.

### Identification of altered pathways

To interpret genome-wide mRNA expression profiles based on biological pathways, we integrated mRNA expression data with the well-documented 218 biological pathways downloaded from the Kyoto Encyclopedia of Genes and Genomes (KEGG) database as of November, 2008 [26]. We defined an average fold-change of expression value of 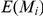 as an indicator of expression changes in biological processes. For a pathway map 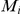 containing a gene set S = {g_1_, g_2_, …, g_k_}, the score of the average differentially expressed level is calculated as follows:
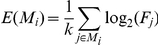



A high 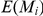 score indicates that a pathway map 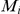 is differentially expressed. The statistic 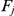 indicates the fold-change in expression of gene *j* between two experimental conditions. To identify the differentially expressed pathways, we calculated the *P-values* representing significant expression changes of identified pathways. We applied a nonparametric permutation test, which estimates the distribution of the statistic 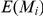 using the permutations of genes or sample labels to compute the *P-values*. We randomly permutated the expression ratio of genes 10,000 times to obtain the null distribution of 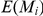 for each pathway map 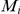 assuming a Gaussian distribution.

### Ranking relevant genes affected by oral TUDCA administration using a PubMed keywords search

To verify the biological relevance of the DEGs, we adopted a gene prioritization method based on PubMed database [27]. As of August, 2009, we performed a PubMed search with the following keywords: ‘fat and liver’, ‘lipid and liver’, ‘triglyceride and liver’, or ‘fatty liver’. Then, we applied the following scoring function to calculate the evidence scores (*ES*) for keyword hits:
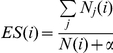
where *ES(i)* is a combined score of the co-citation number of genes *i* with keywords *j*. *N(i)* is the total number of papers related to the gene *i*. *N_j_(i)* indicates the number of papers detected by both gene *i* and keyword *j*. We used a pseudo-count *α* of 10 to account important genes that have more citations. We confirmed that the top ranked genes from this method are related to lipid metabolism by conducting gene enrichment analysis with the BiNGO plugin [28].

## Results

### Effect of TUDCA on hepatic steatosis and glucose homeostasis

**Figure 1:**
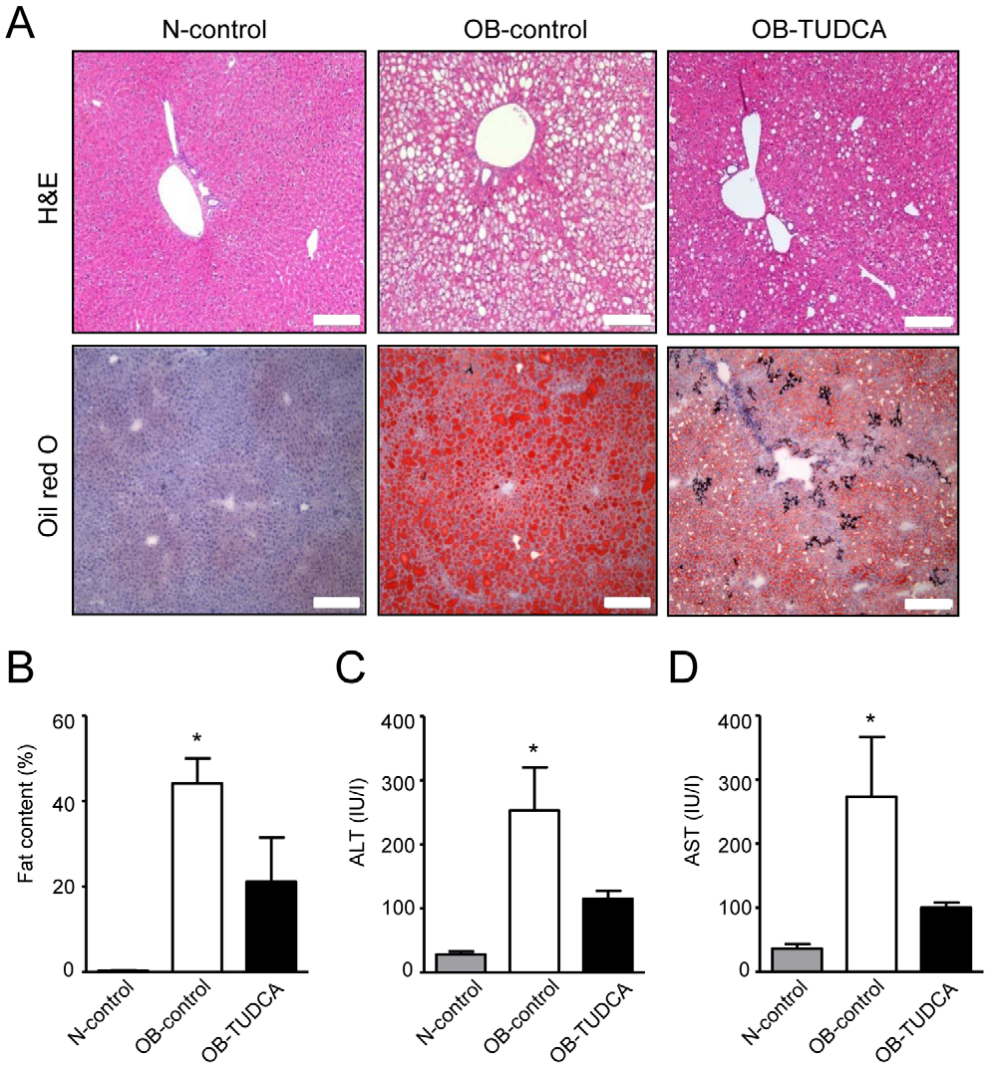
Effects of oral TUDCA treatment on hepatic steatosis in ob/ob mice. (A) H&E and oil red O staining of the liver shows a marked improvement of steatosis. Scale bars indicate 200 µm. (B) Quantified fat content in oil red O stained liver sections was lower in the OB-TUDCA group as compared to the OB-control group. (C, D) Serum ALT and AST levels tended to be lower in the OB-TUDCA group as compared to the OB-control group. In panels B, C and D, * denotes *P*<0.05 compared to the N-control. *P*-values were calculated using the Kruskal-Wallis test and Dunn's multiple comparison test.

**Figure 2:**
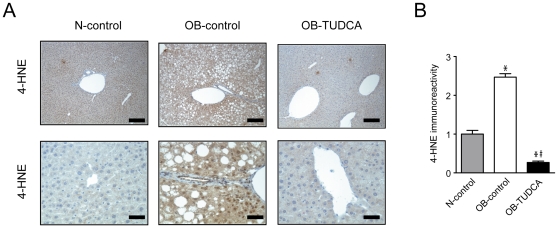
Effects of oral TUDCA treatment on lipid peroxidation. (A) The 4-HNE staining of the liver shows that lipid peroxidation is increased in ob/ob mice, which is reduced by TUDCA treatment. Scale bars in the top and bottom images indicate 200 µm and 800 µm, respectively. (B) Quantification of the intensity of 4-HNE staining. * denotes *P*<0.05 compared to the N-control, and # denotes *P*<0.05 compared to OB-control.

**Figure 3:**
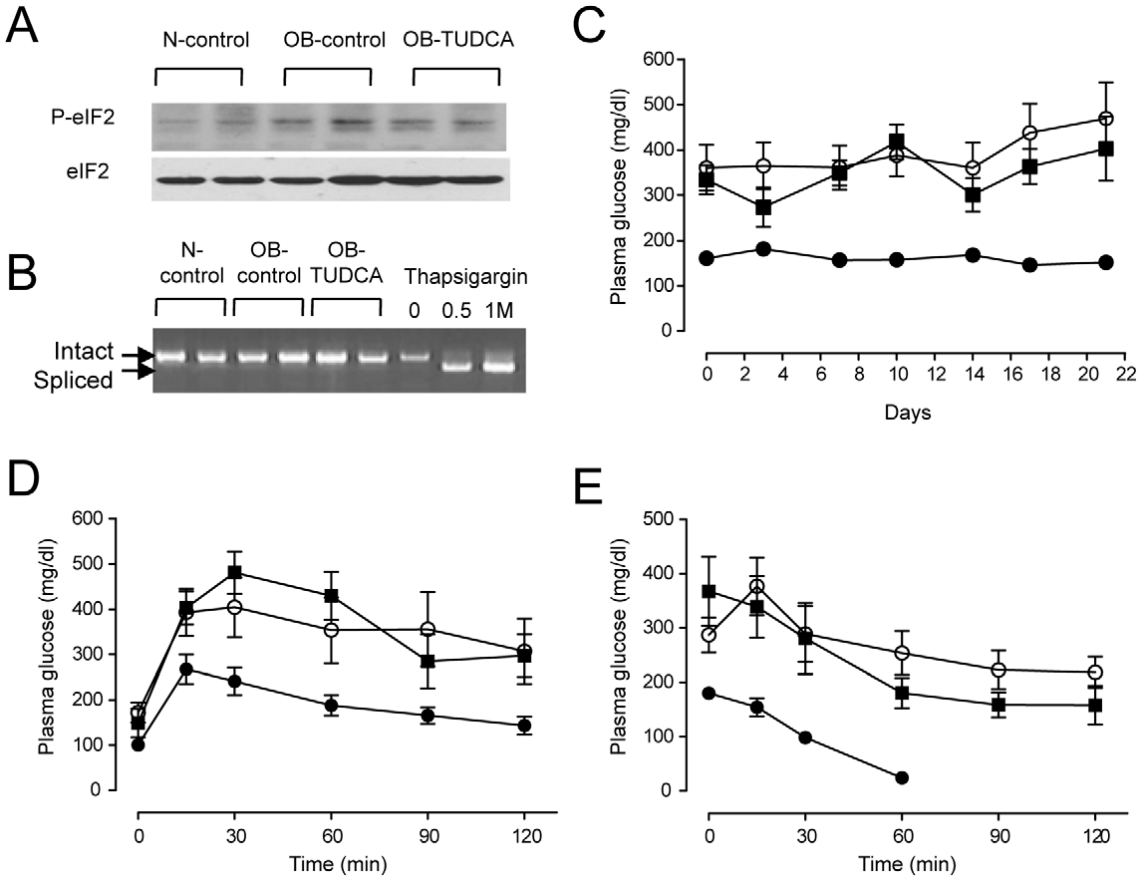
Effects of oral TUDCA treatment on ER stress and glucose homeostasis in ob/ob mice. (A) Phosphorylated eIF2α expression levels are similar among groups. (B) Alternative splicing of *Xbp1* was not detected in any of the three groups. The mRNAs isolated from thapsigargin-treated 3T3 L1 cells were used as controls. (C–E) There was no difference in fed-state blood glucose levels (C), glucose tolerance assessed by IPGTT (D), and insulin sensitivity assessed by ITT (E) between the OB-control and OB-TUDCA groups. Symbols and error bars represent means and SEM, respectively. (•), N-control; (○), OB-control; and (▪), OB-TUDCA mice.

Oral TUDCA treatment markedly improved hepatic steatosis in ob/ob mice as shown by H&E staining (Figure 1*A*). The oil red O stained area approximately exhibited a 2-fold reduction in the OB-TUDCA group as compared to the OB-control group (21.1±10.4% vs. 44.2±5.8%, *P*<0.05) (Figure 1*A* and 1*B*). TUDCA treatment decreased serum ALT and AST levels in the OB-TUDCA group as compared to the OB-control group (Figure 1*C* and 1*D*). Serum triglyceride levels were not significantly different between groups (N-control, 56±7.7 mg/dl; OB-control, 80.0±31.5 mg/dl; OB-TUDCA, 69.3±13.1 mg/dl, *P* = 0.205). The 4-HNE staining was much stronger in the liver of OB-control group than N-control group, which was markedly diminished by TUDCA treatment (Figure 2). There were no differences in expression of the ER stress markers, phosphorylated eIF2α levels (Figure 3*A*) and alternative splicing of *Xbp1* (Figure 3*B*), among the treatment groups. Comparing the OB-TUDCA group with the OB-control group, there were no significant differences in the fed-state glucose levels (Figure 3*C*), glucose tolerance assessed by IPGTT (Figure 3*D*), and insulin sensitivity assessed by ITT (Figure 3*E*) during the study period. In addition, the body weight at day 21 was similar between the OB-control and OB-TUDCA groups (39.6±1.5 g vs. 36.2±0.8 g, respectively; *P*>0.05).

### Altered global gene expression with TUDCA treatment

**Figure 4:**
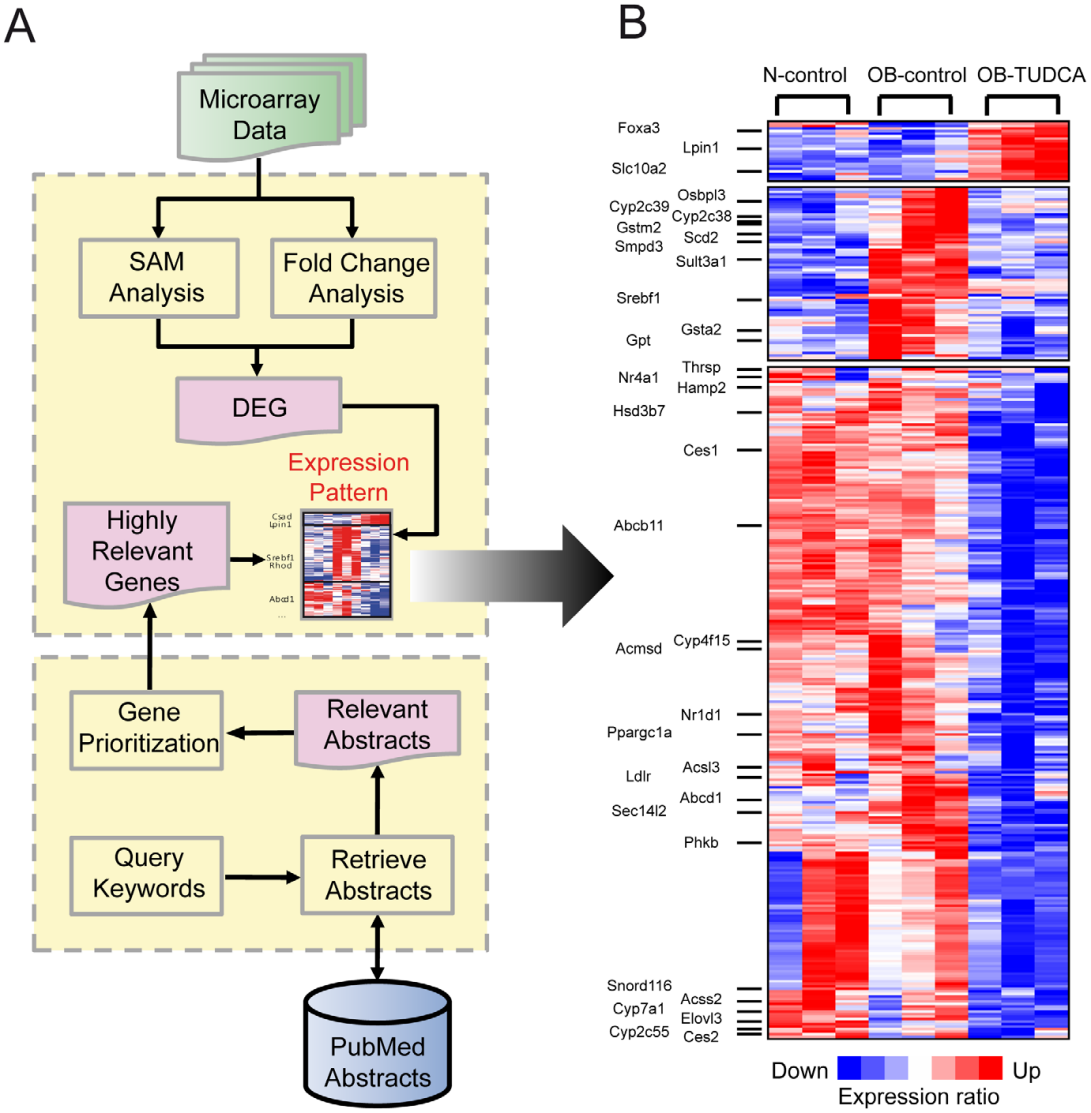
Changes in hepatic gene expression in OB-control and OB-TUDCA groups. (A) Flowchart of DEG selection and relevant gene search. DEGs were selected using SAM and fold-change analysis. The relevant genes were selected using a PubMed database search. (B) The expression matrix shows the relative expression levels of DEGs for each condition. Each column of the matrix represents three experimental conditions: N-control, OB-control, and OB-TUDCA groups. Each row of the matrix represents relative expression data that were gene-wise normalized. Hierarchical clustering was conducted using the following parameters: Pearson correlation and single linkage method.

To examine the effect of oral TUDCA treatment on hepatic gene expression, we compared gene expression profiles of the liver tissue isolated from OB-control and OB-TUDCA mice. First, we assessed the reproducibility of the experiments by comparing the log_2_ signal intensities of microarray data (Figure S1). Scatter plots of log_2_ intensities from paired experiments showed a high level of agreement between the samples (correlation coefficient *r*>0.98). Next, we systematically analyzed the microarray data using various approaches depicted in Figure 4*A*. We identified 357 DEGs (1,199 significantly altered probes) showing high overlap in terms of both fold-change and SAM analyses (Figure 4*B*, Table S1). In the OB-TUDCA group, 334 of 357 DEGs were down-regulated (Table S2). Then, we prioritized the DEGs using evidence scores (*ES*) as described in Materials and Methods. We confirmed that the genes with high *ES* were enriched with the genes regulating lipid metabolism (Table S3; hypergeometric test, *P*<10^−10^). Among the total DEGs, 34 genes were identified as relevant genes (Figure 4*B*, Table S4; hypergeometric test, *P*<10^−9^) and revealed three distinct patterns: (i) over-expressed only in the OB-control group, (ii) over-expressed only in the OB-TUDCA group, (iii) down-regulated only in the OB-TUDCA group (Figure 4*B*). In addition, we validated our microarray data by measuring mRNA levels of representative genes (*Scd2*, *Srebf1*, *Lpin1*, *Pparg*, *Cyp7a1*, and *Abcb11*) using quantitative real time-PCR (Figure S2 and S3).

### Regulation of hepatic lipid homeostasis

**Figure 5:**
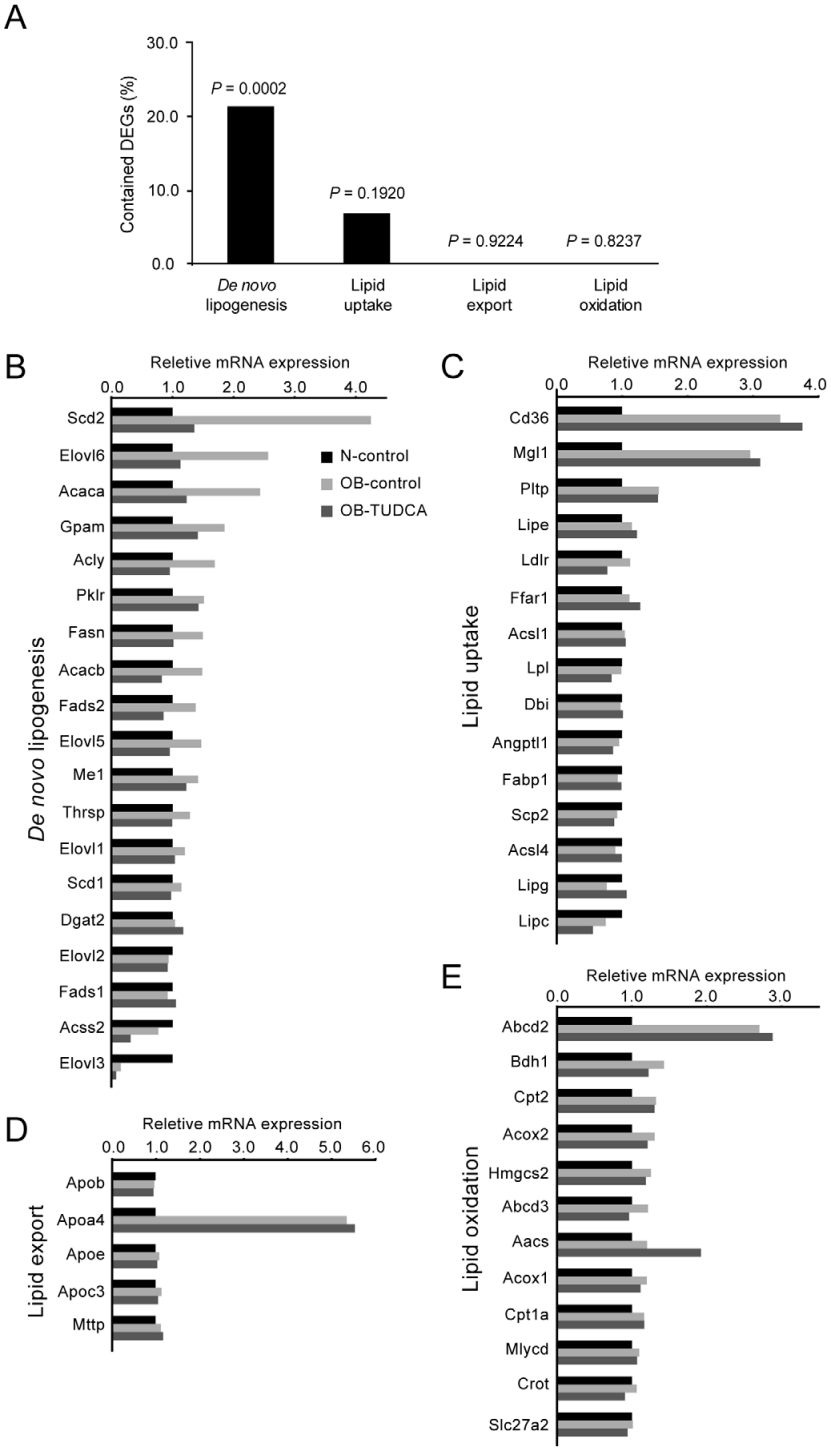
Alteration of gene expression in lipid flux regulating groups. (A) The percentages of included DEGs in each group and its statistical significance. (B–E) Expression changes of genes involved in *de novo* lipogenesis, lipid uptake, export, and oxidation.

To examine which component of lipid homeostasis was altered by TUDCA treatment, we categorized the genes involved in lipid metabolism into four groups (i.e., *de novo* lipogenesis, lipid uptake, lipid export, and lipid oxidation) based on the KEGG pathway and manual curation analyses. As shown in Figure 5*A*, the *de novo* lipogenesis group comprises of the majority of DEGs (hypergeometric test, *P*<10^−3^). Expression levels of genes for fatty acid synthesis (e.g., *Acaca*, *Acacb*, *Acly*, *Gpam*, *Fasn*, *Me1*, *Pklr*, and *Thrsp*) were up-regulated in ob/ob mice but down-regulated with TUDCA treatment (Figure 5*B*). Moreover, the fatty acid elongation and desaturation enzymes (e.g., *Scd2*, *Elovl6*, *Elovl5*, *Fads2*, and *Elovl1*) showed similar expression patterns to the fatty acid synthesis enzymes (Figure 5*B*). In contrast, the expression levels of the genes regulating lipid uptake were generally up-regulated in both OB-control and OB-TUDCA groups. The key lipid uptake genes (e.g., *Cd36*, *Mgl1*, and *Pltp*) were increased in the OB-control mice and their expression levels were not altered with TUDCA treatment (Figure 5*C*). We examined the expression of genes regulating the metabolism of very low density lipoprotein (VLDL), a major pathway regulating hepatic triglyceride export; *Apoa4* was highly up-regulated in ob/ob mice but its expression was not affected by TUDCA treatment. Other VLDL-related genes, including *Apob*, *Apoe*, *Apoc3*, and *Mttp*, showed comparable mRNA expression levels among the three groups (Figure *5D*). The expression levels of fatty acid oxidation-related genes were slightly up-regulated in OB-control mice and their expression levels were not significantly different with TUDCA treatment except for *Aacs*, which showed a further increase with TUDCA treatment (Figure 5*E*).

### Biological pathways affected by TUDCA treatment

**Figure 6:**
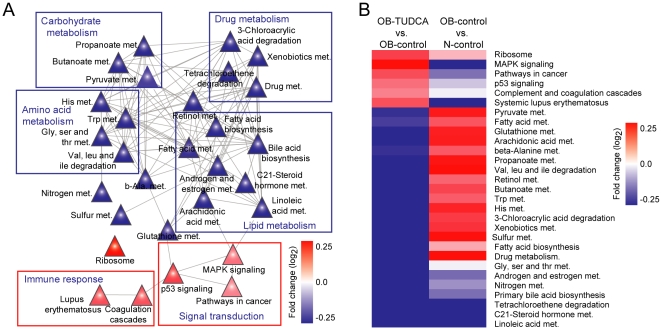
Relationships between altered pathways in the OB-TUDCA group. (A) Abstract view of the pathway-pathway network; it shows the relationship among the pathways by connecting common genes. The colors of triangles represent the average changes in expression of genes involved in the pathway. (B) Average log_2_ fold-change expression pattern of DEPs in N-control vs. OB-control groups and OB-TUDCA vs. OB-control groups. Hierarchical clustering was conducted using the following parameters: Pearson correlation and single linkage method.

In general, genes cooperate with other genes as some parts of pathways that regulate specific biological processes. These associations are of particular importance for relating an altered phenotype with drug treatment at molecular level. To examine the gene expression changes in pathways other than lipid metabolism, we analyzed altered biological pathways in response to TUDCA treatment. We focused on the pathways that were enriched with up- or down-regulated genes and identified 29 differentially expressed pathways (6 up-regulated and 23 down-regulated) (Figure 6*A*, Table S5). Down-regulated pathways in the OB-TUDCA group as compared to the OB-control group included fatty acid biosynthesis, fatty acid metabolism, glutathione metabolism, sulfur metabolism, xenobiotic metabolism, and amino acid metabolism. Most of the altered pathways in this network are extensively connected by common metabolites or genes and display similar expression patterns (Figure 6*A*). Ribosome, inflammatory responses, and cell signaling pathways were up-regulated. Most differentially expressed pathways (DEPs) showed opposite directions of regulation (Figure 6*B*). These results are supported by GO analysis of biological processes involving the identified DEPs (Table 1).

**Table 1:** GO enrichment analysis for biological processes using DEGs in OB-control *vs.* OB-TUDCA mice.

GO-ID	Benjamini *P*-value	# of genes in DEGs	# of genes in whole genome	Description
55114	1.82E-14	40	563	oxidation reduction
8152	4.30E-08	132	6084	metabolic process
8202	4.83E-07	15	141	steroid metabolic process
44255	1.41E-06	27	521	cellular lipid metabolic process
19752	1.41E-06	24	424	carboxylic acid metabolic process
6082	1.41E-06	24	425	organic acid metabolic process
9063	4.62E-06	9	51	amino acid catabolic process
6629	4.62E-06	28	607	lipid metabolic process
9310	1.75E-05	9	61	amine catabolic process
44270	1.75E-05	9	61	nitrogen compound catabolic process
6807	3.84E-04	16	292	nitrogen compound metabolic process
6694	4.13E-04	8	68	steroid biosynthetic process
9308	6.31E-04	15	273	amine metabolic process
6519	8.29E-04	14	247	amino acid and derivative metabolic process

## Discussion

Although the prevalence of NAFLD is alarmingly high, only lifestyle modifications to reduce body weight are generally recommended to treat NAFLD [1], [2], [3]. In this regard, weight-lowering drugs, such as orlistat and sibutramine, were reported to reduce hepatic steatosis and/or serum liver enzyme levels [29], [30]. However, there are no available drugs that have been proven to effectively and safely treat hepatic steatosis independent of weight loss [1]. Thiazolidinediones increase insulin sensitivity and decrease hepatic steatosis and inflammation in humans [11], but they have several side effects including weight gain, heart failure, and increased fracture risk [31]. In the current study, we show that oral TUDCA treatment effectively improves fatty liver disease in ob/ob mice independent of weight loss; this improvement is accompanied by alterations in gene expression, biological processes, and metabolic pathways in the liver.

Although TUDCA is known as a chemical chaperone against ER stress [21], orally administered TUDCA did not alter ER stress markers in the present study. Furthermore, the mRNA levels of ER stress-related genes, such as *Atf4*, *Grp78*, *Trb3* and *Edem*, were comparable among the three treatment groups (Figure S4). Instead, the expression of glutathione S-transferase (Gst) genes (i.e., *Gsta1*, *Gsta2*, *Gsta4*, *Gstm1*, *Gstm2*, *Gstm3*, and *Gstm4*), which are known to increase their expression under oxidative stress [32], were up-regulated in the OB-control group and down-regulated upon TUDCA treatment (Figure S4) implying that oral TUDCA treatment may decrease oxidative stress in the liver. In this regard, TUDCA is known to have antioxidant property that curtails the production of reactive oxygen species [33], [34]. Indeed, we noticed the 4-HNE staining, a marker of lipid peroxidation, was increased in OB-control group compared to N-control group, which was markedly decreased with TUDCA treatment. Of interest, a recent randomized controlled trial revealed that vitamin E, a representative antioxidant available in clinical practice, improves not only hepatic steatosis but also NASH in humans [35]. It is also noteworthy that ER stress does not necessarily accompany fatty liver disease in several independent studies (Table S6). Therefore, we speculate that orally administered TUDCA might improve hepatic steatosis by reducing oxidative stress rather than alleviating ER stress.

Hepatic steatosis results from an imbalance in lipid homeostasis in the liver, where lipid uptake or *de novo* lipogenesis outweighs lipid oxidation or export. The fatty acids in hepatic triglycerides are derived from dietary sources in the form of chylomicron remnants, free fatty acids released from adipose tissue, or from *de novo* lipogenesis [36]. *De novo* lipogenesis accounts for less than 5% in healthy subjects during the postprandial period, while it is known to significantly increase up to 15% in subjects with fatty liver disease [37], [38]. Therefore, enhanced *de novo* lipogenesis is regarded to be a major abnormality of hepatic lipid metabolism in subjects with NAFLD. In mouse models, the knockout of key enzymes involved in lipid synthesis (e.g., *Acc*, *Elovl6*, *Scd1*, *Gpat*, or *Dgat*) reduces hepatic steatosis (reviewed in [39]). Taken together, therapeutic agents targeting a reduction in hepatic *de novo* lipogenesis would be suitable to treat NAFLD patients. In this study, TUDCA markedly reduced hepatic fat content, which was accompanied by a reduction in the expression of *Srebf1* and its down-stream target genes (e.g., *Scd2*, *Elovl6*, and *Acaca*), which is crucial in *de novo* lipogenesis, while other groups of genes involved in lipid uptake, oxidation, and export were not altered (Figure 5). Since *Srebf1* is known to link oxidative stress to hepatic steatosis [40], [41], alleviation of oxidative stress by TUDCA likely decreases the expression of *Srebf1* and subsequently reduces *de novo* lipogenesis. However, TUDCA did not reduce the expression of *Pparg*, which is a critical transcription factor in development of hepatic steatosis in ob/ob mice [42], and its direct target genes (e.g., *Cidec*, *Cd36*, and *Ucp2*), suggesting that the TUDCA effect in improving hepatic steatosis is *Pparg*-independent (Figure S5). Collectively, TUDCA is a promising drug to treat NAFLD, which down-regulates the genes involved in *de novo* lipogenesis.

Although TUDCA substantially reduced hepatic fat content in this study, we could not find any evidence of improved glucose homeostasis. The increased triglyceride content in the liver, the hallmark of NAFLD, is strongly associated with the development of insulin resistance [43], [44], [45]. Although there was no difference in systemic insulin sensitivity assessed by ITT in this study, we cannot exclude the possibility of altered hepatic insulin sensitivity with TUDCA treatment. Our finding is consistent with a recent study in obese human subjects, which demonstrated that 4-week oral TUDCA treatment improved insulin sensitivity in the liver and muscle but did not improve systemic insulin sensitivity and glucose levels [22].

There are a few limitations in this study. (1) Although we administered oral TUDCA twice as much as the dose used in intraperitoneal injection, we could not reproduce improved glucose homeostasis found by Ozcan *et al.* [21]. In this regard, we cannot rule out the possibility that the actual tissue concentration of TUDCA with oral route of administration was lower than that with intraperitoneal administration. We also found that the mRNA expression levels of *Abcb11*, a bile acid transporter [46], and *Cyp7a1*, a bile acid metabolizing enzyme [47], were lower in OB-control group than N-control and were further reduced with TUDCA treatment, which might affect plasma and tissue concentration of TUDCA or other bile acids (Figure S2 and S3). (2) Since the ob/ob mouse is genetically lacking leptin, it may not be an optimal model for common NAFLD in humans. For example, it has been reported that ob/ob mice are resistant to the development of necroinflammation and fibrosis [48]. Hence, our findings need to be confirmed in other animal models such as dietary models and in human subjects. (3) The human equivalent dose corresponding 1,000 mg/kg in mice is ∼80 mg/kg (assuming 60 kg of body weight), which is a higher dose compared to the usual dose of UDCA for primary sclerosing cholangitis (20∼30 mg/kg/day) [49]. Therefore, the safety and efficacy of higher doses of TUDCA should be determined in humans.

In order to identify relevant DEGs and to grasp the dynamic behavior of genes responsible for the recovery of hepatic steatosis by oral TUDCA treatment, we adopted systems biology tools (e.g., a PubMed keywords search and pathway analysis). Using PubMed keywords search, we could validate the selected DEGs based on the preexisting literature dealing with hepatic lipid metabolism. Furthermore, pathway analysis provided us insights to understand the collective behavior of DEGs. We conclude that oral TUDCA administration decreased hepatic lipid content in ob/ob mice by cooperative regulation of genes involved in multiple metabolic pathways, particularly by decreasing the expression of genes regulating *de novo* lipogenesis.

## Supporting Information

Figure S1 Correlation of log_2_ intensities within the same groups. Scatter plots of log_2_ intensities from a pair of experiments within (A) N-control, (B) OB-control, and (C) OB-TUDCA groups. The coefficient of correlation r value is given for each pair.
(0.85 MB TIF)

Figure S2 mRNA expression of Scd2, Srebf1, Lpin1, Pparg, Cyp7a1, and Abcb11. The levels of gene expression were measured by quantitative RT PCR. * denotes P<0.05 compared to N-control, and # denotes P<0.05 compared to OB-control.
(0.44 MB TIF)

Figure S3 mRNA expression of Scd2, Srebf1, Lpin1, Pparg, Cyp7a1, and Abcb11. mRNA expression levels measured by microarray experiments are depicted. * denotes P<0.05 compared to N-control, and # denotes P<0.05 compared to OB-control.
(0.20 MB TIF)

Figure S4 Alterations in mRNA expression levels of genes related to ER stress and oxidative stress.
(0.20 MB TIF)

Figure S5 mRNA expression of Pparg target genes. mRNA expression levels measured by microarray experiments are depicted. * denotes P<0.05 compared to N-control, and # denotes P<0.05 compared to OB-control.
(0.10 MB TIF)

Table S1 Parameters for SAM and Fold-change analysis.
(0.03 MB XLS)

Table S2 List of differentially expressed genes and their fold-changes in expression
(0.06 MB XLS)

Table S3 Results of gene enrichment analysis: the top 500 relevant genes.
(0.04 MB XLS)

Table S4 Results of PubMed keyword search and evidence scores for the top 500 relevant genes.
(0.07 MB XLS)

Table S5 List of altered pathways.
(0.03 MB XLS)

Table S6 List of hepatic steatosis-related literature.
(0.03 MB XLS)

Text S1 (0.04 MB DOC)
