# Supplementary figures and images for: Changes in Hepatic Gene Expression upon Oral Administration of Taurine-Conjugated Ursodeoxycholic Acid in ob/ob Mice
Source: PLoS One. 2010 Nov 5;5(11):e13858. doi: 10.1371/journal.pone.0013858 (PMC2974643; doi:10.1371/journal.pone.0013858)

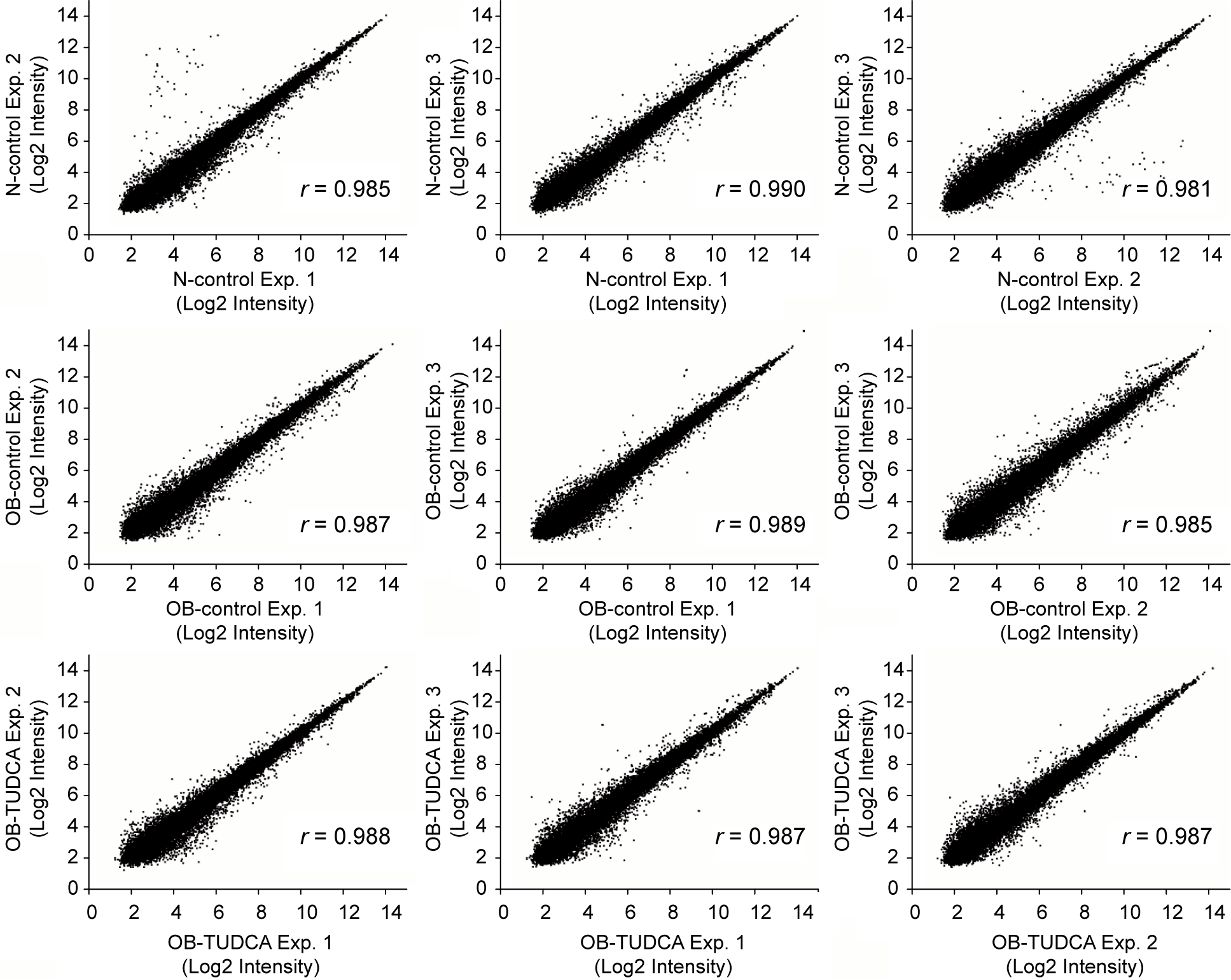

Supplement: Figure S1 — Correlation of log2 intensities within the same groups. Scatter plots of log2 intensities from a pair of experiments within (A) N-control, (B) OB-control, and (C) OB-TUDCA groups. The coefficient of correlation r value is given for each pair. (0.85 MB TIF) [file pone.0013858.s001.tif]

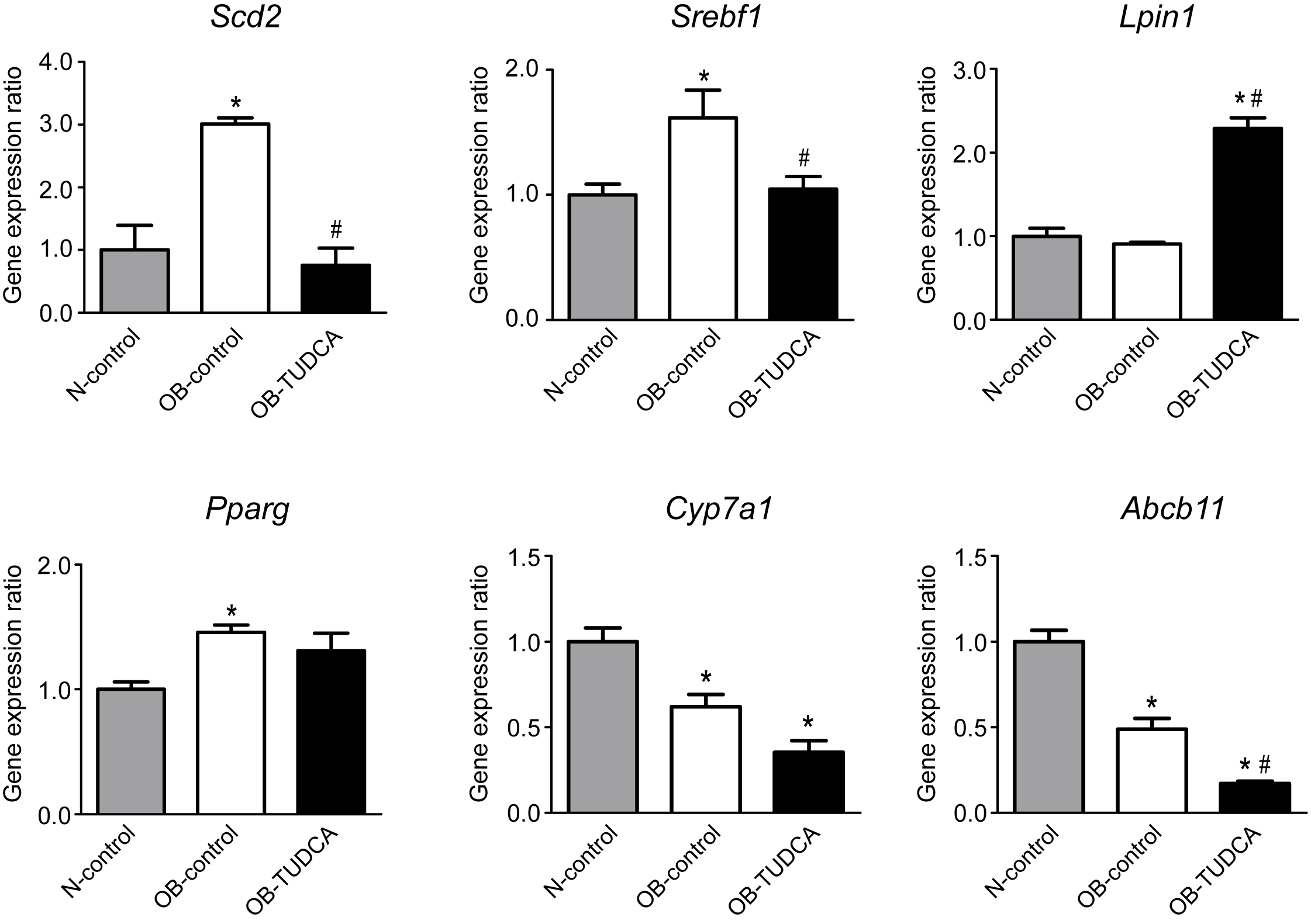

Supplement: Figure S2 — mRNA expression of Scd2, Srebf1, Lpin1, Pparg, Cyp7a1, and Abcb11. The levels of gene expression were measured by quantitative RT PCR. * denotes P<0.05 compared to N-control, and # denotes P<0.05 compared to OB-control. (0.44 MB TIF) [file pone.0013858.s002.tif]

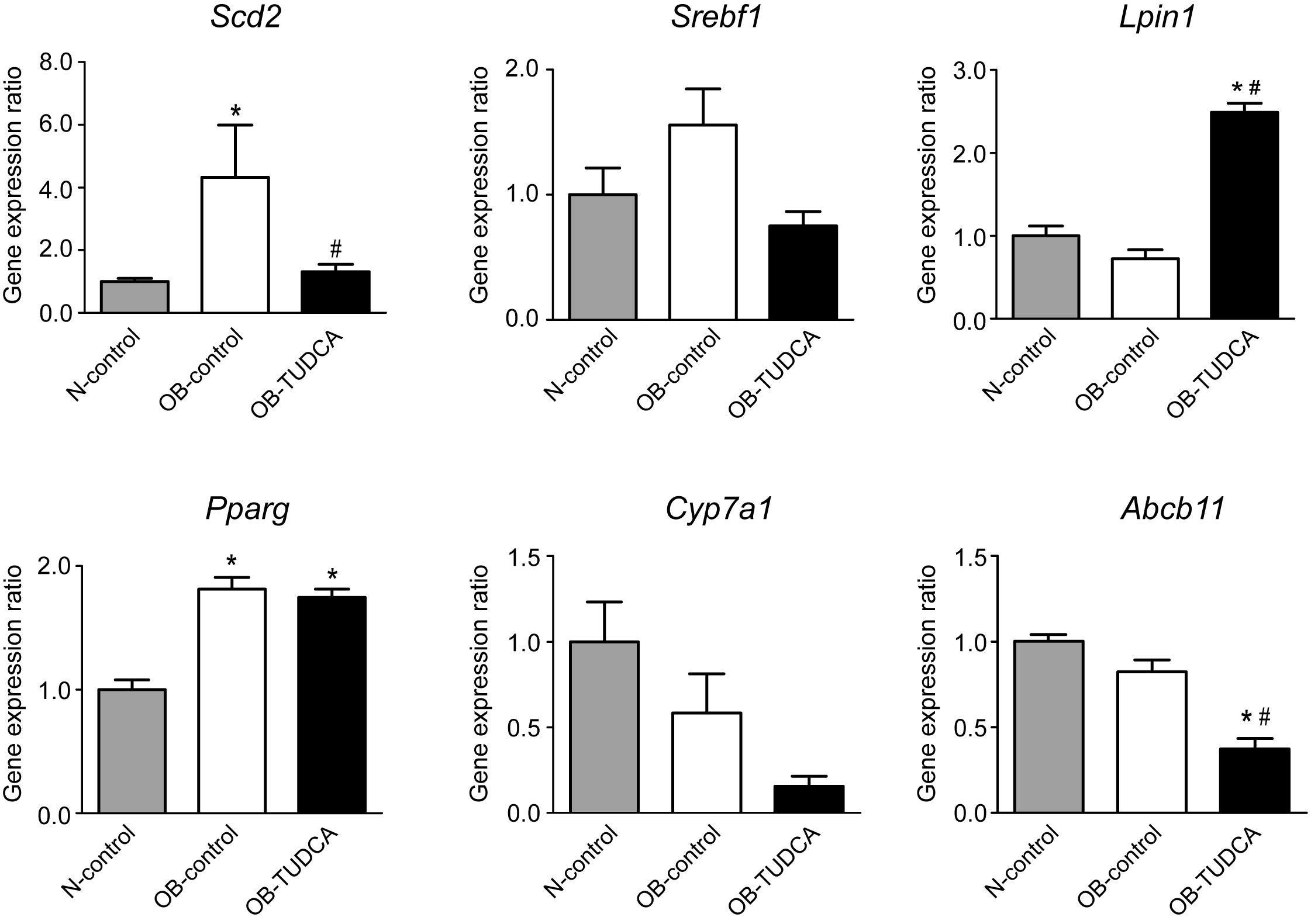

Supplement: Figure S3 — mRNA expression of Scd2, Srebf1, Lpin1, Pparg, Cyp7a1, and Abcb11. mRNA expression levels measured by microarray experiments are depicted. * denotes P<0.05 compared to N-control, and # denotes P<0.05 compared to OB-control. (0.20 MB TIF) [file pone.0013858.s003.tif]

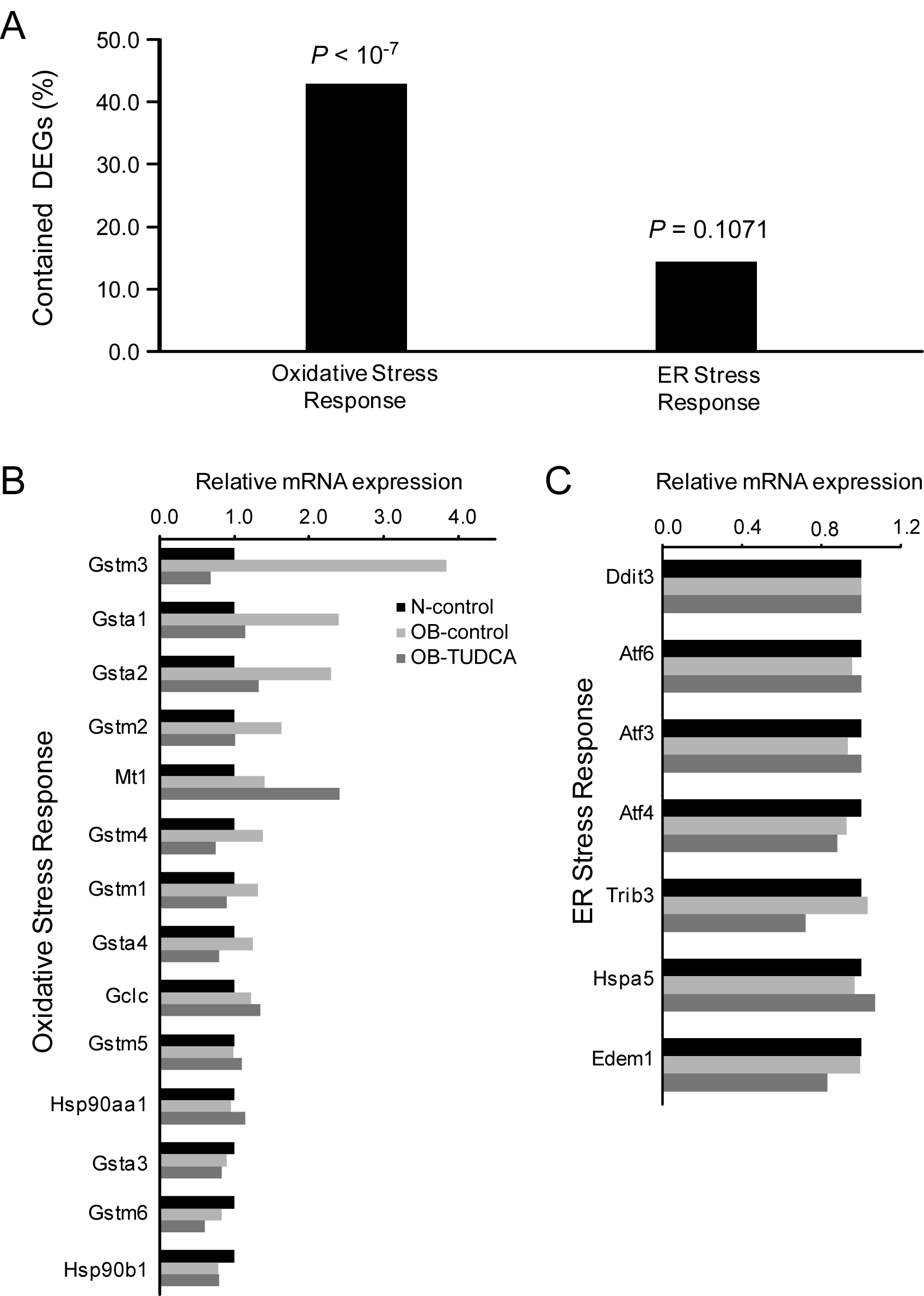

Supplement: Figure S4 — Alterations in mRNA expression levels of genes related to ER stress and oxidative stress. (0.20 MB TIF) [file pone.0013858.s004.tif]

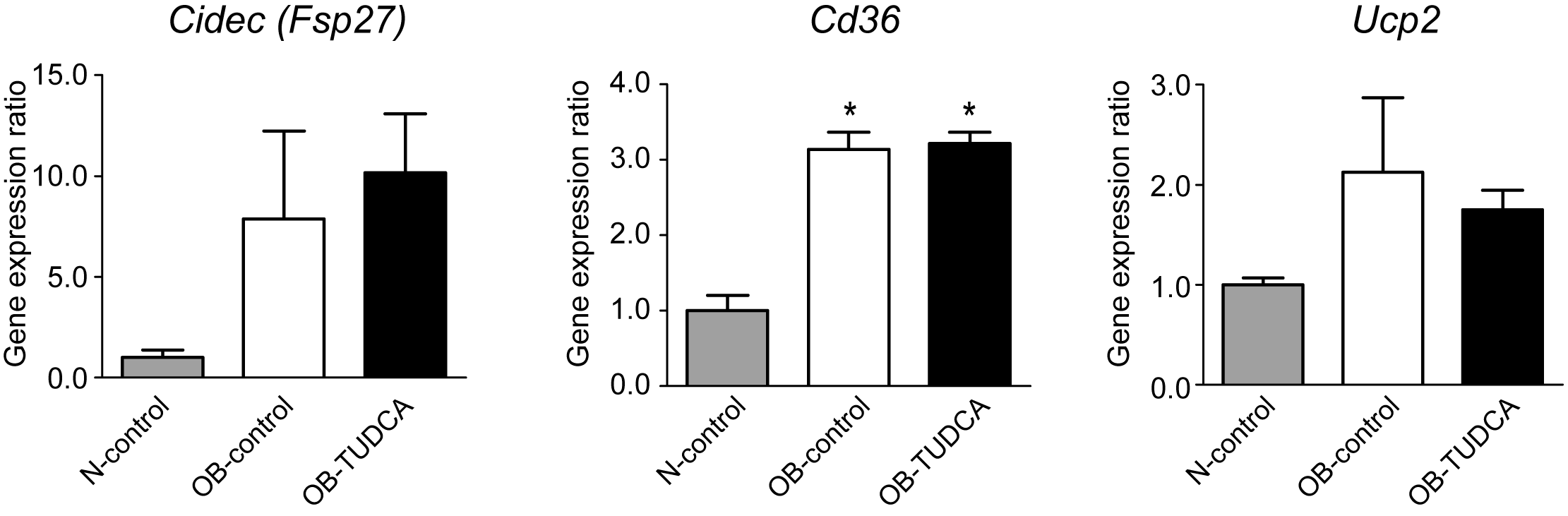

Supplement: Figure S5 — mRNA expression of Pparg target genes. mRNA expression levels measured by microarray experiments are depicted. * denotes P<0.05 compared to N-control, and # denotes P<0.05 compared to OB-control. (0.10 MB TIF) [file pone.0013858.s005.tif]
