# Supplementary material for: Changes in Hepatic Gene Expression upon Oral Administration of Taurine-Conjugated Ursodeoxycholic Acid in ob/ob Mice
Source: PLoS One. 2010 Nov 5;5(11):e13858. doi: 10.1371/journal.pone.0013858 (PMC2974643; doi:10.1371/journal.pone.0013858)
Supplement: Text S1 — (0.04 MB DOC) [file pone.0013858.s012.doc]

**Supplementary Methods**

***Quantitative real-time PCR***

cDNAs, which were prepared using the same method described in the main text, were subjected to quantitative real-time (RT) PCR. The mRNA expression levels of *Scd2*, *Srebf1*, *Lpin1*, *Pparg*, *Cyp7a1*, and *Abcb11* were normalized using the mRNA expression level of *Gapdh*. The probes for each gene (TaqMan® Gene Expression Assays) were purchased from Applied Biosystems (Foster City, CA, USA). Quantitative RT PCR was performed using the TaqMan system (Applied Biosystems) according to the manufacturer’s protocol. All samples were run in triplicate.

***Western blotting***

Liver tissue lysate (50 μg of protein) was electrophoresed using 10% SDS-PAGE, and proteins were transferred onto a nitrocellulose membrane. To detect ER stress, anti-eukaryotic initiation factor 2α (eIF2α) and anti-phosphorylated eIF2α antibodies purchased from Cell Signaling Technology (Beverly, MA, USA) were used for blotting. Hybridized primary antibodies were detected using a horseradish peroxidase-conjugated species-specific IgG antibody. The bands were visualized by enhanced chemiluminescence (Pierce, Rockford, IL, USA).

***Alternative splicing of Xbp1***

Total RNA was extracted from the liver tissue using the RNeasy Mini Kit (Qiagen, Valencia, CA, USA). cDNA was obtained using 1 μg of RNA with oligo-dT20 (Invitrogen, Carlsbad, CA) and avian myeloblastosis virus reverse transcriptase (Invitrogen). cDNA was amplified with a pair of primers (5' GAACCAGGAGTTAAGAACACG 3' and 3' AGGCAACAGTGTTAGAGTTC 5') corresponding to murine *Xbp1* cDNA. Thirty cycles of polymerase chain reaction (PCR) were performed with annealing at 54°C for 30 seconds. PCR products were analyzed using 2.5% agarose gel electrophoresis. RNAs from 3T3 L1 cells treated with various concentrations of thapsigargin were used as positive controls.

***DEG selection and Gene Ontology enrichment analysis***

To identify DEGs, both significance analysis of microarray (SAM) and absolute fold-change were used, as previously described [1,2,3]. We determined the optimal parameters of SAM analysis and the threshold for fold-change by comparing the overlapping DEGs at the probe-level obtained from both analyses. To define the threshold for fold-change, we gradually increased the ratio from 1.5 to 2.5 (1.5, 1.7, 2.0, 2.3 and 2.5) and different delta values of SAM were applied (s_0_ from 0.3 to 3.0). When a single gene matched to the multiple number of probe sets, we took the averaged expression values from the probe sets. To identify the enriched biological functions of DEGs, we performed GO enrichment analysis using the BiNGO plugin (version 2.3) in Cytoscape (version 2.6) [4].

**Supplementary Figure legends and Tables**

**Figure S1. Correlation of log2 intensities within the same groups**. Scatter plots of log2 intensities from a pair of experiments within (A) N-control, (B) OB-control, and (C) OB-TUDCA groups. The coefficient of correlation r value is given for each pair.

**Figure S2. mRNA expression of *Scd2*, *Srebf1*, *Lpin1*, *Pparg*, *Cyp7a1*, and *Abcb11*.** The levels of gene expression were measured by quantitative RT PCR. * denotes *P* <0.05 compared to N-control, and # denotes *P* < 0.05 compared to OB-control.

**Figure S3. mRNA expression of *Scd2*, *Srebf1*, *Lpin1*, *Pparg*, *Cyp7a1*, and *Abcb11*.** mRNA expression levels measured by microarray experiments are depicted. * denotes *P* <0.05 compared to N-control, and # denotes *P* < 0.05 compared to OB-control.

**Figure S4. Alterations in mRNA expression levels of genes related to ER stress and oxidative stress.**

**Figure S5. mRNA expression of *Pparg* target genes.** mRNA expression levels measured by microarray experiments are depicted. * denotes *P* <0.05 compared to N-control, and # denotes *P* < 0.05 compared to OB-control.

**Table S1. Parameters for SAM and Fold-change analysis**

**Table S2. List of differentially expressed genes and their fold-changes in expression**

**Table S3. Results of gene enrichment analysis: the top 500 relevant genes**

**Table S4. Results of PubMed keyword search and evidence scores for the top 500 relevant genes**

**Table S5. List of altered pathways**

**Table S6. List of hepatic steatosis-related literature**

**References**

1. Tusher VG, Tibshirani R, Chu G (2001) Significance analysis of microarrays applied to the ionizing radiation response. Proc Natl Acad Sci U S A 98: 5116-5121.

2. Shi L, Reid LH, Jones WD, Shippy R, Warrington JA, et al. (2006) The MicroArray Quality Control (MAQC) project shows inter- and intraplatform reproducibility of gene expression measurements. Nat Biotechnol 24: 1151-1161.

3. Guo L, Lobenhofer EK, Wang C, Shippy R, Harris SC, et al. (2006) Rat toxicogenomic study reveals analytical consistency across microarray platforms. Nat Biotechnol 24: 1162-1169.

4. Maere S, Heymans K, Kuiper M (2005) BiNGO: a Cytoscape plugin to assess overrepresentation of gene ontology categories in biological networks. Bioinformatics 21: 3448-3449.
